# The challenge of managing mild to moderate distress in patients with end stage renal disease: results from a multi-centre, mixed methods research study and the implications for renal service organisation

**DOI:** 10.1186/s12913-019-4808-4

**Published:** 2019-12-23

**Authors:** Sarah Damery, Kim Sein, Johann Nicholas, Jyoti Baharani, Gill Combes

**Affiliations:** 1 Institute of Applied Health Research, University of Birmingham, B15 2TT, Edgbaston, West Midlands UK; 2 Royal Shrewsbury Hospital, Mytton Oak Road, Shrewsbury, Shropshire, Shrewsbury, SY3 8XQ UK; 3 Renal Unit, New Cross Hospital, Royal Wolverhampton NHS Trust, Wolverhampton Road, Wolverhampton, WV10 0QP UK; 4 Renal Unit, Birmingham Heartlands Hospital, Heart of England NHS Foundation Trust, Bordesley Green East, Birmingham, B9 5SS UK

**Keywords:** Distress, ESRD, CKD, Emotion thermometers, Survey, Qualitative

## Abstract

**Background:**

Lower-level emotional and psychological difficulties (‘distress’) in patients with end stage renal disease (ESRD), can lead to reduced quality of life and poor clinical outcomes. National guidelines mandate provision of emotional and psychological support for renal patients yet little is known about the support that patients may require, or the challenges that staff experience in identifying and responding to patient distress.

**Methods:**

Mixed methods study in renal units at four NHS Trusts in the West Midlands, UK involving cross-sectional surveys of ESRD patients and renal unit staff and semi-structured interviews with 46 purposively-sampled patients and 31 renal unit staff. Interviews explored patients’ experience of distress and personal coping strategies, staff attitudes towards patient distress and perceptions of their role, responsibility and capacity.

**Results:**

Patient distress was widespread (346/1040; 33.3%), and emotional problems were frequently reported. Younger patients, females, those from black and minority ethnic (BME) groups and patients recently initiating dialysis reported particular support needs. Staff recognised the value of supporting distressed patients, yet support often depended on individual staff members’ skills and personal approach. Staff reported difficulties with onward referral to formal counselling and psychology services and a lack of immediate access to less formalised options. There was also a substantial training/skills gap whereby many staff reported lacking the confidence to recognise and respond to patient distress. Staff fell broadly into three groups: ‘Enthusiasts’ who considered identifying and responding to patient distress as integral to their role; ‘Equivocators’ who thought that managing distress was part of their role, but who lacked skills and confidence to do this effectively, and ‘Avoiders’ who did not see managing distress as part of their role and actively avoided the issue with patients.

**Conclusions:**

Embedding the value of emotional support provision into renal unit culture is the key to ‘normalising’ discussions about distress. Immediately accessible, informal support options should be available, and all renal staff should be offered training to proactively identify and reactively manage patient distress. Emotional support for staff is important to ensure that a greater emphasis on managing patient distress is not associated with an increased incidence of staff burnout.

## Background

Patients with end stage renal disease (ESRD) typically experience frequent changes in health status, which give rise to multiple emotional and psychological stressors [1–4], coupled with significant impacts on employment, relationships and lifestyle [5, 6]. There is evidence of widespread lower-level emotional and psychological difficulties (‘mild to moderate distress’) in this patient group, who may experience rates of depression and anxiety substantially higher than in the general population [7, 8]. Untreated emotional and psychological difficulties can be associated with decreased health-related quality of life [9], increased symptom burden [10], and poorer clinical outcomes, including increased healthcare resource use and impaired ability to adhere to challenging dietary and medication regimes [11–13]. Support for patients who experience emotional and psychological difficulties is integral to recommendations regarding the management of long term conditions in general [14], and for patients with renal disease specifically [15]. Both the Department of Health (DH) and National Institute for Health and Care Excellence (NICE) mandate the provision of emotional and psychological support within their national renal guidelines [16, 17]. However, there is little practical guidance as to how such support should be organised, and there is evidence that lower-level support needs often remain unrecognised and untreated [18, 19].

There is a lack of information about the support that patients may require from renal services to manage distress, and there is limited evidence about the factors that may help or hinder staff in identifying and responding to patient distress. Some research has found that renal unit staff find distress hard to recognise [20–22], and studies undertaken in oncology settings suggest that patients may be reluctant to express emotional concerns [23, 24], and that doctors may prioritise clinical over emotional issues during time-pressured consultations [25, 26]. Staff such as nurses and healthcare assistants (HCAs) often develop close relationships with patients over time, and may be well-placed to provide emotional and psychological support. However, their ability to do this effectively may be mediated by issues such as staff education and training [27], perceived role and capacity, confidence, time, knowledge about options for onward referral of distressed patients, and the degree to which managing distress is considered by an individual to be their own responsibility, or the responsibility of others. Existing barriers at the renal unit or individual staff level that prevent staff from being able to effectively manage patient distress would need to be overcome if appropriate interventions are to be developed and targeted towards patients in whom support needs are greatest.

This paper reports the overall findings of a multi-component, mixed methods study that was designed to understand how the recognition and management of renal patients’ emotional and psychological difficulties can be integrated effectively into the ESRD pathway. Papers outlining the detailed findings from each component of the study (the prevalence of patient distress [28]; patient qualitative findings and staff qualitative findings) have been published elsewhere or are in preparation. This paper describes the synthesised findings from the study as a whole. In doing so, it brings together the results from each of the individual study components and presents them according to the key study themes so that recommendations and implications for the organisation of renal services that draw on the findings from each individual study component can be distilled and described in detail.

## Methods

### Aims

The specific aims of the study were to: estimate the prevalence of mild to moderate distress in patients with ESRD; understand how distress may differ for patients at different stages in the ESRD pathway; explore the support that ESRD patients need, want and expect, and to understand the factors that may help or hinder renal staff in managing patient distress.

### Study design and setting

The full methodology is outlined in the published protocol [29]. Briefly, this mixed methods study combined quantitative analysis of data obtained from ESRD patients and renal unit staff via cross-sectional surveys, and qualitative analysis of semi-structured interviews with a purposively sampled selection of patients and staff who had completed the survey. The study was undertaken at renal units in four NHS Trusts in the West Midlands. Sites were selected to achieve maximum geographical spread and ethnic diversity of the patient population, variation in Trust size, rural-urban mix, and in the organisation of emotional and psychological support services for renal patients. The patient work was undertaken in all four study sites; work with renal staff was undertaken in two of the study sites.

### Participants and recruitment

Eligible patients were aged 18 and over, with a diagnosis of Chronic Kidney Disease (CKD) stage 5, and at one of four stages of the ESRD pathway: i) diagnosed with CKD stage 5 and yet to begin renal replacement therapy (RRT); ii) receiving hospital or home dialysis for less than two years; iii) receiving hospital or home dialysis for two or more years, or iv) with a functioning kidney transplant. Grouping dialysis patients on the basis of time since dialysis initiation was hypothesised by the clinicians involved in the study to be important, as distress experience may differ between patients initiating dialysis relatively recently versus those undergoing dialysis for longer. The two year threshold was a pragmatic choice to account for the likelihood of elevated mortality risk for patients in their first year of dialysis treatment, and to ensure that participating patients were more likely to be clinically stable and to have adjusted to their chosen dialysis modality. Patients known to have received psychiatric intervention for distress were excluded. Eligible renal unit staff must have held a managerial or frontline role working with patients at any stage of the ESRD pathway for at least two months prior to study recruitment. Agency and bank staff were excluded from participation.

### Data collection

Eligible patients were sent a postal survey developed for the study, which incorporated numerous validated measures to assess various aspects of distress and emotional adjustment (see Additional file 1). Distress was measured using the Distress Thermometer (DT) and Emotion Thermometers (ET), which scored emotional upset across five domains: distress, anxiety, depression, anger and perceived need for help. The survey also included the Distress Thermometer Problem List which measured the recent experience of specific practical, family, emotional, spiritual and physical problems. Patient adjustment to emotional stressors was measured using the Positive and Negative Affect Schedule (PANAS). Information was also collected on recent events that had caused distress; patients’ perceptions of their ability to cope with their illness and treatment, and selected sociodemographic and clinical information (age, gender, ethnicity, time since diagnosis). Semi-structured interviews were undertaken with a purposive, maximum diversity sample of patients who returned a survey. The topic guide explored issues relating to distress, coping, adjustment and support and patients’ experience of emotional support offered by staff within their renal unit. Survey data collection took place between January 2016 and May 2017; interviews were conducted between April 2016 and May 2017.

All eligible staff in two of the study sites were invited to complete an electronic survey by their clinical lead. The survey focused on respondents’ perceptions of the benefits of identifying and responding to distressed patients; the extent to which they considered providing emotional and psychological support to be part of their role, and how skilled, confident and well-trained they felt in identifying and responding to patient distress. Most questions gave pre-specified answer options based on an 11-point (0 to 10) Likert scale. Data were also collected on age, gender, role, frequency of patient contact, time in post and training attainment. Semi-structured interviews explored staff attitudes, perceptions and perspectives in relation to patient distress; how barriers to staff identifying and responding to distress in ESRD patients could be overcome, and how appropriate changes could be implemented within renal units. Staff were purposively sampled to ensure maximum diversity across job roles. The survey was carried out between February and May 2016; interviews took place between April and December 2016.

### Data analysis

Patients were assigned to one of three distress groups (none to low, mild to moderate, severe) according to their scores on the DT and ET. Patients who scored between 4 and 7 on the DT or between 0 and 3 on the DT and between 4 and 7 on one or more of the ET were assigned to the mild to moderate distress group. All survey responses from patients and staff were analysed descriptively. Interview data were analysed thematically [30], and the qualitative and quantitative findings were synthesised to triangulate themes across the multiple data sources, so that the key messages from the study could be determined.

## Results

### Study participants

The response rate to the patient survey was 27.9% (1040/3730 surveys returned), ranging from 23.0 to 30.4% across study sites. This was lower than anticipated. Most respondents were male (*n* = 635; 60.9%) and in the white ethnic group (*n* = 902; 86.7%). Patients aged between 51 and 69 years old constituted the largest age group (*n* = 441; 42.9%). Nearly two fifths of respondents had received a transplant (*n* = 404; 38.8%) and 28.8% had been on dialysis for two or more years (*n* = 300). Of the 454 patients undergoing regular dialysis, the most common modality was hospital haemodialysis (HD) (*n* = 343; 75.6%). Forty-six patient interviews were undertaken (Table 1). The staff survey response rate was 35.2% (108/307 surveys returned). Nursing staff comprised almost 60% of respondents, with a further 14.8% of responses from doctors. Most respondents were female, and had been performing their current role for 10 or more years. Most respondents (64.8%) reported daily contact with renal patients. Semi-structured interviews were carried out with 31 staff (Table 2).

**Table 1:** Characteristics of patients interviewed

		Site 1	Site 2	Site 3	Site 4	Total
ESRD pathway stage ^a^	Pre-renal replacement therapy	3	3	2	0	8
	Dialysis < 2 years	3	2	0	4	9
	Dialysis 2+ years	1	8	5	1	15
	Transplant	5	8	0	1	14
Gender	Male	6	12	5	5	28
	Female	5	9	2	2	18
Age group	Under 50	2	7	0	1	10
	50–69	4	9	5	4	22
	70+	5	5	2	2	14
Ethnicity	White	7	17	4	5	33
	Black and minority ethnic	4	4	3	2	13
Dialysis type	Hospital haemodialysis	4	9	2	4	19
	Home haemodialysis	0	1	3	0	4
	Peritoneal dialysis	0	0	0	1	1
	TOTAL	12	21	7	6	46

^a^ 46 patients interviewed out of 346 classified as having mild to moderate distress; 346 patients had mild to moderate distress out of 1040 survey respondents; 1040 surveys were returned from 3730 eligible patients surveyed

**Table 2:** Roles of renal unit staff interviewed

Staff roles^a^	Site 1	Site 2	Total
Renal consultant lead	1	1	2
Renal consultant	3	2	5
Dialysis unit nurse manager	0	1	1
Dialysis nurse	5	4	9
Acute ward nurse	0	1	1
Specialist nurse	3	3	6
Renal research nurse	1	0	1
Dietician	2	1	3
Social worker	0	1	1
Occupational therapist	0	1	1
Welfare rights officer	1	0	1
TOTAL	16	15	31

^a^ 31 staff interviewed out of 108 who returned a survey; 108 responses returned from 307 eligible staff who received a survey

### Mild to moderate distress is common in patients with ESRD

One third of patients responding to the survey met the criteria for mild to moderate distress (346/1040; 33.3%). Distress was evident across the renal pathway and was strongly associated with sociodemographic characteristics: prevalence was significantly higher in younger compared to older patients, females compared to males, and in patients from black and minority ethnic (BME) backgrounds compared to patients from the white ethnic group (Table 3).

**Table 3:** Prevalence of mild to moderate distress by sub-group

Variable ^a^	Group	Patients	Prevalence (%)	Comparison of proportions ^b^
ESRD pathway stage	Pre-RRT	64/182	35.2	X^2^ = 4.89; *p* = 0.183
	Dialysis < 2 years	55/154	35.7	
	Dialysis 2+ years	109/300	36.3	
	Transplant	118/404	29.2	
Dialysis type (*n* = 454)	Haemodialysis	129/343	37.6	X^2^ = 3.36; *p* = 0.186
	Home haemodialysis	13/31	41.9	
	Peritoneal dialysis	22/80	27.5	
Age group	< 50 years	78/174	44.8	**X**^**2**^ **= 14.33;** ***p*** **= 0.0008**
	50 to 69 years	145/441	32.9	
	70+ years	119/414	28.7	
Gender	Male	191/633	30.2	**X**^**2**^ **= 6.63;** ***p*** **= 0.01**
	Female	155/407	38.1	
Ethnicity	White	283/902	31.4	**X**^**2**^ **= 10.36;** ***p*** **= 0.0013**
	Black and minority ethnic	63/138	45.7	

^a^ Distress prevalence based on 1040 patient surveys returned out of the 3730 eligible patients surveyed; ^b^ Bold text indicates statistically significant findings

### Distress impacts negatively on patient quality of life and wellbeing

As well as physical problems such as tiredness and pain, emotional problems like worry, sadness and depression were commonly reported by patients. In the qualitative work, interview participants frequently described the emotional burden that ESRD placed upon them (Table 4). Some patients described a loss of identity since their ESRD diagnosis, and noted multiple difficulties in coming to terms with far-reaching changes to their lifestyle and capabilities – because of the physical and emotional burden of ESRD itself, the impacts of diet and fluid restrictions, or (for patients on in-centre haemodialysis in particular), the limitations imposed by the need to plan and undergo dialysis, and to allow for post-dialysis recovery.

**Table 4:** Themes and sub-themes from patient and staff interviews

PATIENTS	
● The emotional burden of ESRD and treatment	
○ Life-saving but not curative treatment	
○ Emotional toll of treatment and impact on lifestyle	
○ Bottling up emotions	
● Patients have complex, multi-faceted support needs	
○ (Presence or absence of) self-support and personal coping mechanisms	
○ Staff recognition of distress	
○ Experience of support provided by renal unit	
○ Experience of support provided by renal staff	
○ Family support	
○ Renal unit atmosphere and environment and impact on disclosing distress	
RENAL STAFF	
● Patients and distress	
○ Identifying distress is challenging (detecting distress; patient reluctance to disclose distress)	
○ Beliefs about distress in ESRD patients (distress at times of change; are some patients more prone to distress?)	
○ Responding to distress is difficult (different approach from clinical care; meeting the needs of BME patients)	
● Staff roles and skills	
○ Role perceptions (it’s everyone’s role; it’s not my role but whose is it?)	
○ Fears (fears related to talking about distress; the emotional load of talking with distressed patients)	
○ Skills, confidence and training (skills; training about supportive services; scepticism over the benefits of training)	
● Care organisation	
○ Limited capacity to respond (perception about how much time is needed; care settings and facilities limit responsiveness; variable access to specialist services)	
○ Differences between staff groups (doctors, nurses and other renal staff; staff groupings)	
● Changes	
○ What helps (staff-patient relationships; more listening, less talking; normalising distress)	
○ What needs to change (access to immediate support; structured approach to identifying distress; reducing the stigma of distress)	

### Who will be distressed and when is unpredictable

The incidence and consequences of distress were influenced by a complex interplay between sociodemographic, treatment-related and individual coping resources/resilience. Specific transitional points in the ESRD pathway may generate particular stressors, such as ESRD diagnosis or initiation of dialysis treatment, but adjustment to ESRD is a dynamic and constant process, and distress may affect any patient at any time. Most interview participants noted that they attempted to maintain a positive attitude towards their condition and the rigours of treatment, and some felt that they had developed effective coping mechanisms. Some of these were influenced or mediated by experiences relating to the renal unit/staff, whereas others related to personal circumstances and individual coping resources (Table 5). Nevertheless, many patients reported mixed feelings about their ability to cope, fear over the possibility that their condition would worsen, and nervousness about the future. These fears were particularly evident in younger patients, who often had to cope with the competing demands of work, family and ESRD treatment, and in BME patients who often reported having a poor understanding of their illness. This could be influenced by language or cultural barriers that inhibited discussions about ESRD and distress with renal staff. There was also a perception that chronic illness was stigmatised within BME communities, and that despite often having strong family networks, patients often felt unable or reluctant to seek emotional support from them.

**Table 5:** Renal unit and individual factors influencing patients’ ability to cope with ESRD

	Influenced by renal unit/staff	Influenced by individual resources
Cognitive reasons	Perceived close relationship with renal staff	Strong personal support network
	Perception of consistent renal unit support	Well-developed self-efficacy
	Feeling well-informed about ESRD and treatment	Positive illness perceptions
	Feeling in control of ESRD and treatment	Ability to recognise unhelpful thoughts
Behavioural reasons	Able to express feelings of distress to staff	Able to discuss emotions with family/friends
	Able to share experiences with other patients	Use of adaptive coping techniques
	Effective coping strategies developed with support from the renal unit	Able to sustain family and social relationships
	Gained confidence from increased knowledge and understanding about ESRD and treatment	Able to maintain hobbies, activities and interests

### Patients want help from the renal unit and specific patient groups expressed a particular need for support

Not all patients with ESRD are distressed; not all distressed patients want support, and not all distressed patients who want support necessarily want this to be provided by their renal unit. A number of interview participants believed that coping with distress was their own responsibility and was not something that required support from renal staff. It must also be noted that experiencing some degree of distress should be considered a normal response to having a chronic disease. However, younger patients, females, patients from BME groups and those who had recently begun dialysis treatment had the highest rates of distress reporting, reported the most significant degree of impairment in their ability to cope, and the highest self-reported levels of support need.

### Patients may be reluctant to disclose distress

Providing effective support for distressed patients requires that renal staff can identify patients who may need help, and that staff have the capacity and capability to help patients manage their distress. However, many patients reported that they deliberately hid distress to avoid burdening healthcare staff who were perceived as being under stress themselves, too busy carrying out clinical tasks, or with limited time to discuss emotional issues. Some patients also felt that talking about emotions with staff on the renal unit was inappropriate, believing that staff would not understand their situation or that they lacked the relevant skills to handle any emotional issues raised. Patients who attended the renal unit infrequently – such as those on home therapies or who had received a transplant – reported limited opportunities to raise emotional issues, which added to their reluctance to disclose distress when they did attend the renal unit.

### Staff may find patient distress difficult to recognise

Staff participants recognised that providing emotional support to patients had intrinsic value and felt that it was an important component of high quality care. However, healthcare professionals may be relatively poor at recognising the signs of distress in their patients, especially when patients normalise their feelings, or go out of their way to ‘bottle them up’. Key barriers to renal staff being able to identify distress were related to: a) renal unit organisation, b) time, c) training/skills, and d) perceptions of responsibility.

Identifying distress often depended on an individual staff member’s personal interest, skills and individual approach to patients, rather than emotional support being considered integral to ESRD patient care. The organisation of care in outpatient clinics and dialysis units meant that staff did not always see the same patients, making it difficult to recognise distress as staff did not always know patients well. Patients often noted that the deliberately jovial and upbeat atmosphere created by staff within the renal unit inhibited discussions about distress as this atmosphere gave the impression that negative emotions should not be expressed. Further to this, heavy workloads and the need to prioritise clinical rather than emotional issues were noted by some staff as a barrier to discussing distress with patients.

Renal staff who had received training in how to handle distressed patients were more likely to feel that this was part of their role, and these staff members typically reported a significantly greater level of confidence in managing patient distress effectively. Other staff members who had not developed key skills, either due to a lack of training or a lack of long-term experience in working with renal patients, often described feeling less able to recognise and interpret non-verbal signs of distress; not knowing how to ask patients about their emotions, and feeling unable to ‘contain’ distress so that dealing with a distressed patient did not take up a disproportionate amount of time. As a result, some staff felt that managing emotional issues required a specialist skillset, the lack of which was often cited as a reason for avoiding the proactive identification of patient distress for fear that the ‘floodgates would open’, without the capacity to provide acceptable or appropriate support. This was particularly pertinent for clinical staff, who are typically trained to offer solutions when faced with (clinical) problems, and who felt an expectation from patients that distress should be managed in the same way. However, the patient interviews showed that many patients who disclose emotional issues to renal staff are not necessarily seeking a solution, but simply want to be listened to in an empathetic and sympathetic way. This suggests that there may be some fundamental differences in what patients and staff consider effective support to entail, and that the avoidance of emotional issues by some staff due to the perception that they are unable to provide a solution may be based on a largely unfounded view about what many patients expect.

Although many staff considered identifying and responding to patient distress as being part of everyone’s role in the renal unit (including non-clinical staff), they recognised that this was rarely the case in practice. Some staff felt that distressed patients should self-identify as distressed rather than distress being something that staff should actively look for. Others felt that dealing with distressed patients was the responsibility of staff members with specialist skills, or that it was optional, based on an individual’s personal inclination to include this in their role. A number of staff deferred to other staff members. For example, there was some evidence that consultants felt that nurses should take primary responsibility for dealing with distressed patients as they were thought to have the most appropriate skills for doing so. This view was not always shared by nurses, who felt in many cases that patients’ emotional concerns could be most effectively dealt with during consultations with doctors.

Staff views about responsibilities for the management of patient distress were often underpinned by fears related to their role (particularly for doctors), the extent to which they felt comfortable talking about emotional issues, and the impact that managing patient distress might have on themselves as individuals. For example, most doctors reported tensions between dealing with (potentially solvable) medical issues and dealing with (difficult to solve) emotional issues, and often felt that discussing emotional issues with patients compromised their role as a doctor by impairing their ability to remain detached and objective. Other staff said that although they were able to identify distress in patients, they were unlikely to deal with it because they did not feel comfortable talking about feelings in general, or with patients. Finally, many staff expressed concerns over the emotional impact that dealing with patient distress would have on themselves. Some felt that discussing patients’ emotional issues was made more challenging when they had their own problems to deal with, leaving them without the emotional capacity to take on the additional burden of patients’ problems.

### Staff may lack the capacity and capability to provide appropriate support

Even if renal unit staff are able to identify patient distress, they may lack the capacity and capability to provide appropriate support. This may be because they lack the training and skills to facilitate the provision of support, or because of the way that support services are organised and accessed. For example, many staff noted having little information about appropriate support options they could offer to patients. Furthermore, onward patient referral to renal psychologists, counselling or community services was seen as challenging given the need to wait for referrals to be processed and for appointments to become available. This was seen as particularly problematic given that many patients wanted immediate support at the time of need. The formality of support options was also seen as important: there was a sense from many patients that referral to formal psychological services could be stigmatising, since formalising support also formalises distress. Indeed, patients were most likely to report a preference for support options that focused on discussing emotional issues during face-to-face meetings with renal doctors or nurses. Thus, having a wider range of less formal options for managing distress may be effective and acceptable to patients.

Overall, staff fell broadly into three groups. First, the ‘enthusiasts’ who thought that identifying and responding to patient distress was an intrinsic part of their role. These individuals were proactive in identifying distress and had developed the skills and confidence to do so through experience. Second, ‘equivocators’ considered managing patient distress to be theoretically part of their role, but in practice felt that they lacked the skills and confidence to do this effectively. Third, ‘avoiders’ who thought that dealing with patient distress was a key element of care, but who did not see this as part of their own role. These staff actively avoided the issue of distress with patients, prioritised clinical care over emotional wellbeing and tended to see staff other than themselves as being better placed to manage patient distress.

## Discussion

Despite the lower than expected response rate to the patient and staff surveys undertaken as part of this study, our findings suggest that there may be substantial levels of unmet need amongst ESRD patients, and that despite the provision of emotional and psychological support services being mandated within national renal guidelines, a gap exists between the high levels of patient distress observed in this study and the low levels of active distress management by renal units and staff. Bridging this gap is unlikely to be achievable through simple interventions – patient distress often has complex roots and influences, and staff experience numerous challenges in being able to identify distress and respond effectively. The complexity of these issues suggests that any modifications to service organisation and delivery would need to be multi-faceted [31], encompassing changes at both the organisational (unit) and individual (staff) levels to improve patient education, enhance staff skills, implement changes to available support options, and facilitate changes to renal unit culture.

First, there is scope for improved education for patients about the distress that they may experience at different stages of the ESRD pathway. Renal patients typically receive a substantial amount of pre-RRT education. This is partly due to the fact that – unlike some other chronic conditions with an unpredictable onset – patients with renal failure can usually be supported throughout their initial period of deterioration, allowing a relatively incremental adjustment to life on dialysis [19]. Consequently, patient education typically stops after dialysis treatment has been successfully initiated. There may be scope for continuing patient education throughout the ESRD pathway [32], even after successful transplantation. Effective education may entail the provision of practical, factual information about treatments and prognosis, and information about signposting to the full range of support services that may be available. There is also scope for patient education to become more effective in managing patients’ expectations about the distress they may experience as their condition progresses, ensuring openness and full disclosure of the negative issues that may arise. This approach has been found in other studies to be effective in reducing distress and improving general wellbeing [33, 34]. As well as providing factual information and education about the potential for distress to ESRD patients, effective support should take a proactive approach towards equipping patients better to cope with the distress they may experience [35, 36]. Building emotional resilience into patient education could help patients to release stressful emotion, develop coping skills and facilitate healthy emotional responses [37]. Informal, educational drop-in sessions on building resilience and coping could be given periodically by renal psychologists, counsellors or by specialist nurses and may offer the additional benefit of ‘normalising’ discussions about distress.

A lack of staff training in identifying and handling patient distress was commonly cited as a barrier to the provision of appropriate and effective support. Staff who had received training in how to identify and manage patient distress were more likely to feel that this was their responsibility rather than that of other professionals, and were confident in their ability to handle distress effectively. The overall goal of training would be to facilitate both the proactive identification of patient distress, and the appropriate reactive skills once distress has been identified. Having visible senior leadership behind any changes to training and skills development would be beneficial, as would the formalisation of emotional support provision into the renal pathway at the renal unit level. There may be the most potential to make progress if training interventions were initially offered on a targeted basis to the ‘equivocator’ staff group, who felt that managing patient distress should be integral to their role, but felt that they lacked the skills and confidence to do so effectively. The ‘enthusiasts’ were already skilled and confident in their ability to identify and manage patient distress, and it is likely that these staff would benefit from having more personal support and opportunities for debriefing their work with patients. The ‘avoiders’ may not benefit greatly from skills development opportunities in the short-term, as they did not see emotional support provision as part of their role and substantial groundwork may be needed at the renal unit level to convince these staff to change.

Staff training could include education focusing on patient-centred communication strategies, such as the use of open-ended questions and empathetic statements [38]. Recognising that distress can manifest itself in many ways, training could also focus on recognising the verbal and non-verbal signs that indicate a patient may be distressed; how to identify distress quickly; case scenarios about handling difficult situations, and learning from the experience of other professionals such as counsellors and psychologists. Staff training in identifying and handling patient distress ‘in the moment’ through the use of empathy and sympathetic listening may be effective in the short-term for many patients, particularly as patients often reported that they did not necessarily want a solution to their problems but may simply want to unburden themselves of their emotional issues. At the same time, it is important to ensure that a greater emphasis on renal unit staff taking a proactive role in identifying and managing patient distress is not associated with a greater likelihood of either staff burnout (i.e. exhaustion caused by work demands) or ‘compassion fatigue’ (i.e. the personal costs over time of caring for patients who are suffering). It is important that staff are able to place ‘boundaries around their interactions with distressed patients to ensure that providing support does not compromise a staff member’s own mental health. A number of studies have found clear links between healthcare professionals’ sense of personal wellbeing and their ability to provide good quality patient care [39, 40]. Evidence from oncology settings suggests some benefit to staff from the provision of emotional support based on regular group meetings or peer support to enhance personal coping resources [41, 42].

For patients who need further information or onward referral, it is important that a series of options are available and that renal units have appropriate pathways for patient referral or signposting to additional support. This relies on staff members having clear, practical information about distress that they can pass on to patients. Renal units could provide drop-in sessions and immediately accessible in-house emotional support services that do not require formal referral such as embedded specialist nurses with counselling expertise who could provide emotional support at the time of need. For transplant patients, or those on home-based dialysis modalities, having a named nurse as a patient’s direct contact could be effective in allowing the disclosure of emotional concerns to become normalised and managed in a timely manner.

Research has found a consistently positive association between healthcare setting culture and patient outcomes [43]. The changes proposed following this study would require a change to renal unit culture to ensure that distress is seen as a normal part of ESRD and is discussed routinely with patients, who are encouraged to seek help and to develop appropriate coping skills. Culture change would make the identification and management of patient distress central to renal unit activities, and create an environment for patients and staff where talking about distress is normalised and emotional support valued. Embedding a culture which places greater emphasis on the value of providing emotional support to patients would also benefit the ‘equivocator’ and ‘avoider’ staff groups by demonstrating the centrality of emotional support as a key element of effective patient care.

## Limitations

This study had a number of limitations which may limit its generalisability. Responses to the patient and staff surveys were relatively low (27.9 and 35.2% respectively). All participants in the qualitative research were recruited from amongst the survey respondents, which may have introduced some bias into the sample, as the characteristics of patients who responded to the survey and/or afreed to be interviewed may have differed from those who declined. However, interview participants were purposively sampled to ensure maximum diversity according to key sociodemographic and clinical characteristics for patients, and in terms of job role for renal staff. Nevertheless, the low response rates seen in this study compromise its wider generalisability despite the internal validity of the study being high. A further limitation was the cross-sectional rather than longitudinal research design, which allows limited understanding of the relationship for patients between time since ESRD diagnosis and ability to cope with the resulting stressors. Finally, although the inclusion of multiple study sites is likely to have minimised any bias that may have arisen from variations in the organisation of renal services, the staff survey and interviews were only undertaken at two of the four study sites. This may have limited our ability to understand some of the mediating factors associated with the local context of individual renal units that may have improved or inhibited staff members’ ability to identify and respond to patient distress.

## Conclusion

This large, multi-site study is the first to explore the prevalence of mild to moderate distress in patients with ESRD and has identified considerable room for improvement in the way that renal services are organised and support provided to distressed patients. Mild to moderate distress is common and there are potentially substantial unmet support needs within the ESRD patient population, with younger patients, females, and patients from BME communities particularly affected. There is unlikely to be any single intervention that can support patients with distress – its incidence is largely unpredictable and its duration uncertain. However, changes to all levels of renal unit organisation and to the way that individual staff manage their patients will be required if the identification of, and response to patient distress is to be optimised.

## Supplementary information

**Additional file 1.** Survey used to collect quantitative data from patients with end-stage renal disease.

## Data Availability

The dataset generated and analysed during the current study is not publicly available due to ethical concerns over the possibility that individual study participants could be identified from their data. The dataset is available from the corresponding author on reasonable request.

## Abbreviations

BME: Black and minority ethnic
CKD: Chronic kidney disease
DH: Department of health
DT: Distress thermometer
ESRD: End stage renal disease
ET: Emotion thermometers
HCA: Healthcare assistant
HD: Hospital haemodialysis
NHS: National health service
NICE: National institute for health and care excellence
PANAS: Positive and negative affect schedule
RRT: Renal replacement therapy
